# Modeling the Dynamic Transmission of Dengue Fever: Investigating Disease Persistence

**DOI:** 10.1371/journal.pntd.0000942

**Published:** 2011-01-11

**Authors:** Líliam César de Castro Medeiros, César Augusto Rodrigues Castilho, Cynthia Braga, Wayner Vieira de Souza, Leda Regis, Antonio Miguel Vieira Monteiro

**Affiliations:** 1 Centro de Ciência do Sistema Terrestre, Instituto Nacional de Pesquisas Espaciais, INPE, São José dos Campos, Brazil; 2 Departamento de Matemática, Universidade Federal de Pernambuco, UFPE, Recife, Brazil; 3 Departamento de Parasitologia, Centro de Pesquisa Aggeu Magalhães, CPqAM, Recife, Brazil; 4 Departamento de Saúde Coletiva, Centro de Pesquisa Aggeu Magalhães, CPqAM, Recife, Brazil; 5 Departamento de Entomologia, Centro de Pesquisa Aggeu Magalhães, CPqAM, Recife, Brazil; 6 Divisão de Processamento de Imagens, Instituto Nacional de Pesquisas Espaciais, INPE, São José dos Campos, Brazil; Yale University, United States of America

## Abstract

**Background:**

Dengue is a disease of great complexity, due to interactions between humans, mosquitoes and various virus serotypes as well as efficient vector survival strategies. Thus, understanding the factors influencing the persistence of the disease has been a challenge for scientists and policy makers. The aim of this study is to investigate the influence of various factors related to humans and vectors in the maintenance of viral transmission during extended periods.

**Methodology/Principal Findings:**

We developed a stochastic cellular automata model to simulate the spread of dengue fever in a dense community. Each cell can correspond to a built area, and human and mosquito populations are individually monitored during the simulations. Human mobility and renewal, as well as vector infestation, are taken into consideration. To investigate the factors influencing the maintenance of viral circulation, two sets of simulations were performed: (1^st^) varying human renewal rates and human population sizes and (2^nd^) varying the house index (fraction of infested buildings) and vector per human ratio. We found that viral transmission is inhibited with the combination of small human populations with low renewal rates. It is also shown that maintenance of viral circulation for extended periods is possible at low values of house index. Based on the results of the model and on a study conducted in the city of Recife, Brazil, which associates vector infestation with *Aedes aegytpi* egg counts, we question the current methodology used in calculating the house index, based on larval survey.

**Conclusions/Significance:**

This study contributed to a better understanding of the dynamics of dengue subsistence. Using basic concepts of metapopulations, we concluded that low infestation rates in a few neighborhoods ensure the persistence of dengue in large cities and suggested that better strategies should be implemented to obtain measures of house index values, in order to improve the dengue monitoring and control system.

## Author Summary

Dengue is the most rapidly spreading mosquito-borne viral disease in the world and approximately 2.5 billion people live in dengue endemic countries. In Brazil it is mainly transmitted by *Aedes aegypti* mosquitoes. The wide clinical spectrum ranges from asymptomatic infections or mild illness, to the more severe forms of infection such as dengue hemorrhagic fever or dengue shock syndrome. The spread and dramatic increase in the occurrence of dengue cases in tropical and subtropical countries has been blamed on uncontrolled urbanization, population growth and international traveling. Vaccines are under development and the only current disease control strategy is trying to keep the vector quantity at the lowest possible levels. Mathematical models have been developed to help understand the disease's epidemiology. These models aim not only to predict epidemics but also to expand the capacity of phenomena explanation. We developed a spatially explicit model to simulate the dengue transmission in a densely populated area. The model involves the dynamic interactions between humans and mosquitoes and takes into account human mobility as an important factor of disease spread. We investigated the importance of human population size, human renewal rate, household infestation and ratio of vectors per person in the maintenance of sustained viral circulation.

## Introduction

Dengue is currently the most important arthropod-borne disease, affecting around 50 million people worldwide every year, mostly in urban and semi-urban areas [1]. During the last decades, the disease has spread to most tropical countries and has become an important cause of death and hospitalizations by dengue hemorrhagic fever and dengue shock syndrome [2]. South-east Asia is one of the most affected regions, where dengue hemorrhagic fever is a leading cause of morbidity and death among children [1]. In the Americas, a significant increase in dengue incidence has been observed in the last two decades [3].

Dengue can be caused by four distinct but antigenically related serotypes which are mainly transmitted by *Aedes aegypti* mosquitoes. The wide clinical spectrum ranges from asymptomatic infections or mild illness, to the more severe forms of infection such as dengue hemorrhagic fever and dengue shock syndrome. Infection by one serotype produces long-life immunity to that serotype but does not protect against infection by others [4].

A wide variety of factors influence the spatial and temporal dynamics of mosquito populations and, therefore, dengue transmission patterns in human populations [5]. Temperature, rainfall and humidity interfere in all stages of vector development from the emergence and viability of eggs, to the size and longevity of adult mosquitoes, as well as their dispersal in the environment [6]–[13]. Additionally, factors such as unplanned urbanization, high human population density [14], the precariousness of garbage collection systems and water supply [15], [16] - frequent problems in developing countries - favor the proliferation of breeding sites and infection spread.

While the development of dengue vaccines is still underway [17], [18] and assuming that mosquito eradication is a remote possibility, the only alternative of controlling dengue transmission remains in keeping the vector population at the lowest possible levels [19], [2]. However, the threshold has not been established yet [20].

For dengue control programs to be effective, information on the occurrence of infection and disease in the population are essential. However, as most dengue infections are asymptomatic or unapparent, presenting themselves as non-differential fevers of unknown etiology, surveillance systems based on the monitoring and notification of symptomatic cases have low sensitivity and are not capable of detecting low or sporadic transmission [2], [21].

Mathematical and statistical models have been developed in order to provide a better understanding of the nature and dynamics of the transmission of dengue infection, as well as predict outbreaks and simulate the impact of control strategies in disease transmission [22], [16]. Most of these approaches are based on ordinary differential equations or statistical models without exploring the spatial pattern of disease transmission; e.g. [23]–[26]. A summary of the approaches used up to 2006 was reviewed by Nishiura [27]. More recently, models have been developed which incorporate the spatial structure of dengue spread [28]–[32], as well as models that use complex networks [33], [34].

Another class of models used to investigate the disease transmission process is that of cellular automata (CA) [35]–[39] which are self-reproductive dynamic systems, where time and space are discretized [40]. They are composed of a finite regular lattice of cells, called cellular space, each one with an identical pattern of local connections to other cells, and subjected to given boundary conditions [39], [41]. Each cell can assume a state, among a finite set of states, which can change at every time-step according to local transition rules (deterministic or stochastic) based on the states of the cell and of its neighbors. Models based on cellular automata have the advantage of being spatially explicit in the sense that their elements can be individually tracked in space through which the simulations are carried out. They constitute a class of spatio-temporal dynamics models that allow the development of a virtual environment that creates and explores different scenarios of the dynamics of disease. CA-based models have been used to study the dynamics of dengue fever [42], [43], [34]. Santos et al. [42] considered the immature forms of *Aedes aegipty* in their model to study the patterns of dengue in Salvador city, in the Northeastern coast of Brazil. Ramchurn et al. [43] and Silva et al. [34] used a combination of cellular automata and scale free network ideas to map the evolution of dengue fever.

We propose a stochastic cellular automata model that simulates dengue transmission in a hypothetical population, aiming to perform a qualitative analysis of factors that influence disease transmission. Unlike the mathematical models based on differential equations, the proposed CA-based model of diffusion of dengue fever uses heterogeneous rules for human mobility. The role of human mobility in the transmission of infectious diseases has been previously investigated [44]–[46], including in dengue epidemics [32]. This article investigates the influence of factors related to both humans (renewal rate and population size) and vectors (house infestation index, vector density per human and biting frequency) in the maintenance of viral circulation for extended periods. The approach was based on the urban shape of the populous *Brasilia Teimosa* neighborhood within the city of Recife, Brazil. Previous surveys have found high *Aedes aegypti* infestation rates [19] and prevalence of dengue seropositivity higher than 90% in this area [47].

## Methods

The proposed model takes into account existing knowledge about the biological cycle and disease transmission of dengue infection in humans and vectors. Although the model supports the assumption of co-circulation of all serotypes, in this initial approach the simplest scenario which considers the circulation of only one serotype was simulated. Some parameters of the model are constant while others follow a probability distribution (Table 1). In this model, human population is not age-structured and vertical transmission and climatic variability are not considered. The development and implementation of the model were carried out using MatLab, version 6.5.

**Table 1:** Parameters set individually for each cell and for each individual (human or vector) and for unit of time.

Cell Parameters			
Parameter	Symbol	Description	Assumption
Number of humans in cell *(i,j)*	*N_h_(i,j)*	Gaussian distribution with average 4 and deviation 2	To ensure that 68% of occupied cells will have between 2 and 6 people
Vector-human ratio in cell *(i,j)*	*N_vh_(i,j)*	Uniform probability density function within the interval [0,*max_v_*]	To be proportional to the number of humans in cell *(i,j)*
Number of vectors in cell *(i,j)*	*N_v_(i,j)*	Defined *by int(N_vh_(i,j) · N_h_(i,j))* +*u*;where *u* = 1, with probability equal to the decimal part of *N_vh_(i,j) · N_h_(i,j),* and *u* = 0, otherwise	

Individual Parameters			
Parameter	Symbol	Description	Assumption
Intrinsic incubation period (days)	*τ_i_*	Gaussian distribution with average 5.5 and deviation 1.5	To ensure that 68% of cases will be between 4 and 7 days
Infective period in humans (days)	*τ_vir_*	Gaussian distribution with average 4.5 and deviation 1.5	To ensure that 68% of cases will be between 3 and 6 days
Extrinsic incubation period (days)	*τ_e_*	Gaussian distribution with average 9 and deviation 0.25	To ensure that 95% of cases will be between 8.5 and 9.5 days

Individual Parameters by Unit of Time		
Parameter	Symbol	Description
Number of bites of mosquito *k* on day *t* in cell *(i,j)*	*b_v_(i,j,k,t)*	Defined by *int(b_fv_)* +*u*;where *u* = 1, with probability equal to the decimal part of *b_fv_,* and *u* = 0, otherwise

### Assumptions of the model

#### Dengue infection in humans

Infected human individuals are not contagious during the intrinsic incubation period that ranges between 4.5 and 7 days, with a small probability of exceeding 10 days [20]. The viraemia occurs at the end of the incubation period and lasts approximately 4 or 5 days, although it might take up 12 days [20]. During this period, the infected individuals are infective for the vector. Following the viraemia period, the infected individuals become immune for the same serotype, assuming recovery for that specific serotype.

#### Vector behavior and transmission cycle

*Aedes aegypti* rarely fly long distances, reaching a maximum of 25 meters in urban environments and being centered on the house where they breed [48], [49]. Although movement towards the nearest houses may be intense, its dispersal is usually limited to a small area [50], suggesting that people rather than mosquitoes are the primary mode of dengue virus dissemination within and among communities [1], [51]. Only female mosquitoes bite hosts and their movements are essentially done for searching food, shelter, mating opportunities and oviposition sites [49].

These vectors feed almost exclusively on humans [52]–[54]. If a susceptible vector bites an infected person during the viraemic period, it may become infected and subsequently transmit the virus to other healthy humans after an extrinsic incubation period of 8 to 12 days [1], [4]. Once infected, the female *Aedes* mosquito carries the virus during its life span [1]. *Aedes aegypti* vectors are very nervous feeders, disrupting the feeding process at the slightest movement, only to return to the same or a different person to continue feeding moments later. Due to this behavior, *Aedes aegypti* females often feed on several persons during a single blood meal and, if infectious, may infect multiple persons in a short time [4]. It is assumed that the survival rate of *Aedes aegypti* females is not dependent on their age [55], the vector's averaged life span is assumed to be 40 days [56] and that there is a 90% chance that a bite of an infected mosquito transmit dengue virus to a susceptible individual [55], [57]. We found no information about the transmission probability from human to vector and assumed 90% for this value.

**Figure 1:**
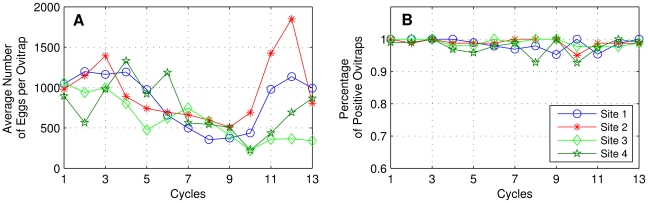
Ovitraps experiment in a survey conducted in the city of Recife, Brazil. Research conducted from April 2004 to April 2005, counting 13 cycles of 28 days each, in four urban areas with the presence of *Aedes aegypti* mosquitoes. (**A**) Average number of *Aedes aegypti* eggs per ovitrap per site. Legend in B. (**B**) Percentage of ovitraps with *Aedes aegypti* eggs inside, for each site. Despite the control intervention implemented from December 2004 to April 2005 in sites 1 and 3 (corresponding to cycles 9-13 and to dry season), the percentage of ovitraps with *Aedes aegypti* eggs showed no major changes (part B). Data modified from Regis et al. [19].

With relation to the spatial distribution of vectors, based on a survey conducted in the city of Recife using geo-referenced ovitraps [19], we assumed that the house index patterns of *Aedes aegypti* are normally very high year-round (above 90%), whereas the total amount of vectors shows seasonal variability. Figure 1 shows the results of this research: while the number of eggs varies, the percentage of positive ovitraps virtually does not change after application of control measures (sites 1 and 3). The number of vectors per house also varies between studies. For example, a study in Puerto Rico found 5–10 *Aedes aegypti* females per residence [58], whereas in Thailand it was estimated an average of 20 females per room in each house [59].

### The Spatially Explicit Transmission (SET) Model

**Figure 2:**
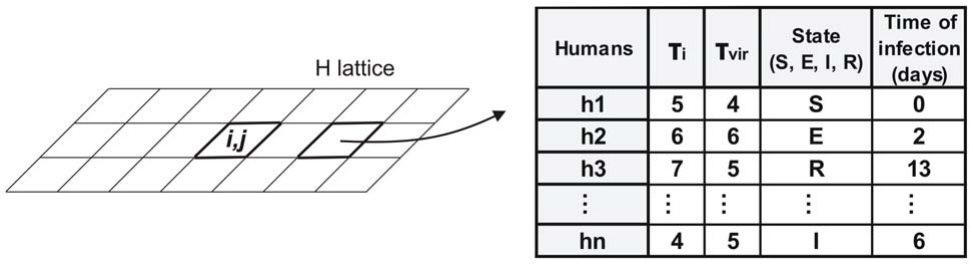
Illustration of the information stored in a non-empty cell of the *H* lattice.

The CA-based model consists of two bidimensional square lattices, *H* and *M*, both of same size and spatial location, representing the spaces occupied by humans and mosquitoes, respectively. Each cell of *H* and *M* corresponds to a lot that can be occupied by a building or be empty. The probability of a lot being occupied by humans is *ρ_h_*. Each cell with position *(i,j)* that contain humans is represented by a matrix, named *H(i,j),* where information related to the humans living in the existing building (intrinsic incubation period (*τ_i_*), period of infectivity (*τ_vir_*), status of the individual in relation to disease and infection time) are stored. Figure 2 illustrates the information stored in a non-empty cell *H(i,j)* of the *H* lattice.

Assuming that *Aedes aegypti* are usually located in the places where humans reside, the model states that a percentage *ρ_v_* of non empty cells in the *H* lattice is infested by mosquitoes. This percentage - called house index (HI) – represents the proportion of mosquito-infested buildings. The model considers only *Aedes aegypti* females. The population of female vectors in each cell is a function of the number of humans and the vector/human ratio within the corresponding cell in *H* lattice. The vector/human ratio varies from building to building, following a uniform distribution in the interval [0,*max_v_*], where *max_v_* is the maximum number of vectors for each human assumed in the model.

**Figure 3:**
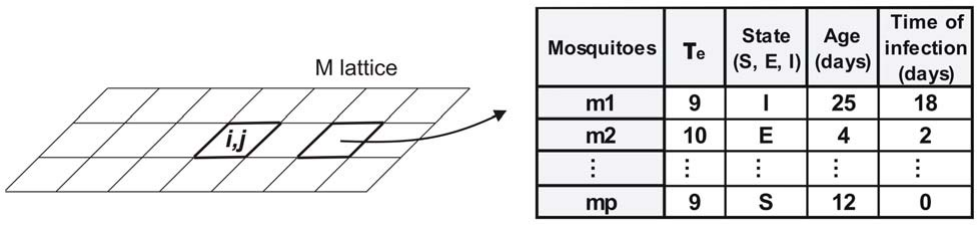
Illustration of the information stored in a non-empty cell of the M lattice.

In the *M* lattice each cell of position (*i,j)* which contain mosquitoes is represented by a matrix *M(i,j)* that contains information on the existing vector population in the corresponding building. The matrix *M(i,j)* contains the following information pertaining to each mosquito: the extrinsic incubation period (*τ_e_*), the age of the vector, the state of the mosquito in relation to the disease and the time of infection. Figure 3 illustrates the information in a non empty unit in the *M* lattice.

At the beginning of each simulation, the model generates an initial configuration for the H and M lattices, assuming that the entire population (humans and vectors) are susceptible, except for a single randomly chosen infected human. For this initial configuration, the following parameters in each cell are pre-determined: (1) the human population (*N_h_(i,j)*); (2) the vector population (*N_v_(i,j)*); (3) the intrinsic incubation periods and infectivity periods for each human and (4) the extrinsic incubation periods for each vector. These parameters are summarized in Table 1. The values assigned to individual parameters *τ_i_*, *τ_vir_* and *τ_e_* are in agreement with literature [20], [4], [48], [55], [60].

The dynamics of human-mosquito interactions is based on the following rule: every day each mosquito randomly selects one or a few humans to bite, according to a daily frequency of bites *b_fv_* (number of blood meals per day). Contact between humans and mosquitoes can occur two ways: local and global contact. The local contact is determined by the search strategy of mosquitoes for human targets which reside nearby. The global contact is determined by the movement of humans, which may come from elsewhere and visit buildings where mosquitoes are found.

During the process of interaction between humans and mosquitoes, each human can assume one of four states with respect to each serotype: susceptible (S), exposed (E), infectious (I) or recovered (R) and each vector can assume one of three states with respect to each serotype: susceptible (S), exposed (E) and infectious (I). The duration of the exposed state (infected but not infectious) corresponds to the incubation period. If there is a contact between a susceptible human and an infectious vector, the human may become exposed with probability *β_vh_*. On the other hand, if an infectious human has contact with a susceptible mosquito, the latter becomes exposed at a probability *β_hv_*.

The human population was modeled considering a single annual renewal rate (*ρ_nh_*), as a combination of births, deaths, immigration and emigration. All newcomers are assumed to be susceptible to the dengue virus. The total amount of humans and mosquitoes was kept constant during all simulations. Mosquito survival rate is assumed to satisfy a Poisson distribution.

The boundary conditions are periodic, which means that opposite borders of the lattice are connected to each other to form a toric topology [61]. Each time step corresponds to one day. The constant parameters of the model are: Human population size (*N_h_*), percentage of human occupation (*ρ_h_*), house index (*ρ_v_*), maximum ratio of vectors per human (*max_v_*), mosquito daily biting frequency (*b_fv_*), vector daily survival probability (*p_s_*), transmission probability from human to vector (*β_hv_*), transmission probability from vector to human (*β_vh_*), annual human renewal rate (*ρ_nh_*) and mobility parameters. The parameters that vary by cell, individual or per unit of time are described in Table 1, wherein function *int(·)* means the integer part of.

#### Target choice by mosquitoes

**Figure 4:**
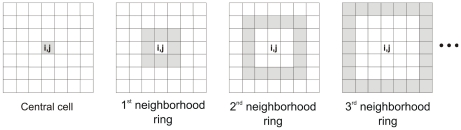
Configuration of the neighborhood rings of cell *(i,j)*.

The model also assumes that the probability that vectors bite humans decreases as the distance from its cell of origin increases. The random selection of the target-cell by the mosquito depends on its flight range *R*, that is defined *a priori.* The cell in which the mosquito resides is called central cell, while the set of neighboring cells adjacent to the central cell is named first neighborhood ring. The set of adjacent cells (external) to the first neighborhood is named second neighborhood ring, and so on. In this model, the flight range of mosquitoes indicates how many neighborhood rings the vector can travel in search of a human target. Some neighborhood rings of a generic cell are illustrated in Figure 4.

At first, without considering human movements in the model, for every mosquito a random human target is chosen in three steps: (1^st^) Draw of a neighborhood ring, according to the vector of predetermined probabilities *r* = (*r_0_*, *r_1_*, …, *r_R_*), where *r_0_* >*r_1_* > … >*r_R_* e *r_0_* +*r_1_* + … +*r_R_* = 1; (2^nd^) Uniform random selection of a occupied cell in the chosen neighborhood ring; (3^rd^) Uniform random selection of a human inside the cell drawn in the previous step.

#### Human Mobility

We consider random human mobility in which a daily percentage of the human population leaves its residence and randomly chooses other buildings to visit. Movements can be homogeneous (assuming that all households have the same characteristics) or concentrated in public locations. The daily rate of human mobility (*ρ_mob_*) defines the fraction of people that can visit other cells every day. Individuals which are both infectious and symptomatic are assigned a mobility of zero, as they remain at home or in hospital. If *I_hi_(t)* is the number of infectious humans at time *t*, then the percentage of individuals who are infectious at time *t* is *I_hi_(t)/N_h_*. If *ρ_ass_* is the percentage of infectious who are asymptomatic, then the percentage of humans who are both infectious and symptomatic at time *t* is
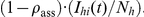



As these humans do not leave their homes during their infectious periods, only the fraction
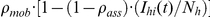
of people will move to visit other places. Among these, a percentage *ρ_mobCom_* will move to public locations, which comprise a proportion of *ρ_Com_* of the buildings in the *H* lattice, whereas the fraction (1− *ρ_mobCom_*) will visit residences. The visited cells may have mosquitoes with probability *ρ_v_* (here *ρ_v_* coincides with house index). The mosquitoes that are in the visited cell can bite the visitor with probability
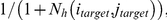
where *(i_target_,j_target_)* is the position of the site chosen by mosquito for its blood meal and *N_h_(i_target_,j_target_)* is the amount of humans in cell *(i_target_,j_target_)*. Thus, the probability that a vector bites a visitor at time *t* is
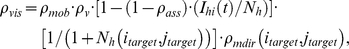
where *ρ_mdir_(i_target_,j_target_)* is defined by *ρ_mobCom,_* if *(i_target_,j_target_)* is a public location; and (1-*ρ_mobCom_)*, if *(i_target_,j_target_)* is a domestic site.

In the simulations that there are no public locations in the cellular space (*ρ_mobCom_* = 0), the probability that a vector bites a visitor in each cell *(i_target_,j_target_)* at time t is simply
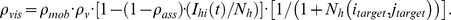



With human mobility taken into account, the third stage of the choice of target by the mosquito changes to:

(3^rd^) with probability *ρ_vis_*, randomly select a visitor of any cell of the *H* lattice; if that is not possible, select uniformly a human in cell *(i_target_,j_target_).*

For public locations, the maximum number of vectors per human was calculated assuming that there is a fixed amount of people in these places that spends the whole day on this site. This amount is based on the same rule for de *N_h_(i,j)* in Table 1.

#### Human Renewal Rate

Considering the initial dengue-naïve open population in the sense that human renewal is taken into account, after a dengue epidemic, the small number of susceptible individuals in addition to the births and immigration of new healthy individuals allows the maintenance of viral transmission, despite low rates. Through time, the number of susceptible humans increases until it is sufficient to initiate a new outbreak. This is a classic framework that helps to understand the periodicity of epidemics [62]. We assume only positive or zero rates of human renewal. Theoretically, the daily number of humans being replaced by new susceptible ones would be 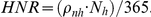



However, the SET model approaches the daily number of renewed humans at time *t* by 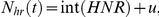
where *u* = 1, with probability equal to the decimal part of *HNR,* and *u* = 0, otherwise.

#### Active Viral Transmission

To investigate the values of the minimum parameters required for maintenance of viral transmission for extended periods, two sets of simulations were performed with the number of iterations corresponding to seven years. Our tests showed that this period is sufficient for steady-state establishment.

For both sets of simulations, the stochastic parameters used are those shown in Table 1 and the fixed parameters are given in Table 2, while the parameters which differ in the two simulations (both fixed and varying) are given in Tables 3 and 4. The percentage of asymptomatic patients was chosen according to field surveys conducted in the city of Recife, by the Aggeu Magalhães research center (CPqAM; unpublished). The probability of daily survival of the mosquitoes was chosen so that their average life was 40 days. The maximum number of vectors to humans in each building was selected based on [63] and on the experience of the CPqAM entomologists.

**Table 2:** Fixed parameters used to investigate the maintenance of viral transmission.

Constant Parameters		
Parameter	Symbol	Values
Percentage of human occupation	*ρ_h_*	0.9
Transmission probability from human to vector	*β_hv_*	0.9
Transmission probability from vector to human	*β_vh_*	0.9
Mosquito daily survival probability	*p_s_*	0.983
Neighborhood selection probabilities	*r*	(0.7, 0.3)
Percentage of asymptomatic infected humans	*ρ_ass_*	0.65
Overall rate of human mobility	*ρ_mob_*	0.5
Mobility rate to public locations	*ρ_mobCom_*	0.9
Percentage of public locations	*ρ_Com_*	0.05

**Table 3:** Parameters used to investigate the maintenance of viral transmission in the first set of simulations.

Parameter	Symbol	Values
Human population size	*N_h_*	{2000, 4000, …, 12000}
Annual human renewal rate	*ρ_nh_*	{1, 2, …, 6} %
House index	*ρ_v_*	0.9
Maximum ratio of vectors per human	*max_v_*	2
Mosquito daily bite rate	*b_fv_*	{1, 1.5}

**Table 4:** As in Table 3, but for the second set of simulations.

Parameter	Symbol	Values
Human population size	*N_h_*	8,000
Annual human renewal rate	*ρ_nh_*	5%
House index	*ρ_v_*	{0.5, 2, 5, 10, 20, 30, 50, 70, 90} %
Maximum ratio of vectors per human	*max_v_*	{0.5, 1, 2}
Mosquito daily bite rate	*b_fv_*	{1, 1.5}

At first, we simulated the spread of dengue infection varying human parameters (population sizes and renewal rates), while the other model parameters remained fixed. Based on the observed data of the *Brasilia Teimosa* neighborhood in Recife, where a high density of *Aedes aegypti* eggs was registered, with positivity in more than 90% of the homogeneously distributed traps during the entire year [19], we assumed a high vector infestation in the simulations, within 90% of the buildings. For each combination of variable parameters in Table 3, we performed simulations with 50 replications, for which we recorded the cases showing viral transmission (epidemic and the maintenance of viral transmission) in the first six months. Among the recorded cases, we calculated the percentage of replications in which viral transmission remained active year after year, until the seventh year.

With the results of the first set of simulations, we fixed the size of the human population and human renewal rate to values which ensure a high chance of maintaining viral transmission for extended periods. Then we performed the second set of simulations, varying the house index and vector per person ratio, in order to investigate the values that are able to eliminate viral transmission. For each combination of variable parameters in Table 4, we performed simulations with 200 replications, from which we recorded the cases showing viral transmission in the first six months. Among the latter cases, we counted the percentage of replications which viral transmission was sustained year after year until the seventh year. The difference in the number of replications for each set of simulations (for Table 3 and for Table 4) is due to computational limitations in the first experiment, whose simulations are time consuming due to high house index considered, especially for larger human populations.

## Results

### The behavior of the compartmental model

**Figure 5:**
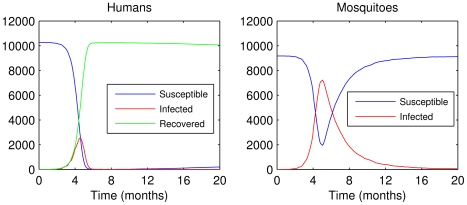
The behavior of the compartmental model for humans and mosquitoes.

Figure 5 shows the evolution of the SEIR framework for humans and the SEI pattern for mosquitoes. Here, as well as in subsequent figures, we considered for simplicity the infected state as the sum of the individuals of exposed or infectious state at time step *t*. Also, we considered a neighborhood with approximately 10,000 inhabitants and house infestation index of 90%. This agrees qualitatively with the patterns of compartmental epidemiological models [39], .

### Human Mobility

To study the effects of human movement, we conducted simple experiments with different mobility configurations. The stochastic parameters used are those shown in Table 1 and the fixed parameters are given in Table 2. Other constants were: population size of 10,000, annual human renewal rate of 0%, house index of 70%, mosquito daily bite rate of 1 and maximum ratio of vectors per human of 2.

**Figure 6:**
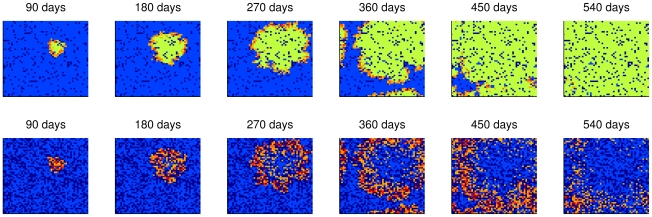
Spread pattern of dengue infection in a simulation without human mobility. Spread of infection for humans (top) and for mosquitoes (bottom). Color legend in Table 5.

**Figure 7 pntd-0000942-g007:**
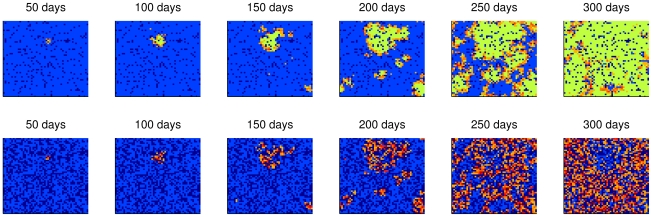
Spread pattern of dengue infection considering concentrated human mobility. Every day 50% of the population leaves its home. Among these, 90% of them go to public locations (which corresponds to 5% of the cells) while the remainder visits other domiciles. Spread of infection for humans (top) and for mosquitoes (bottom). Color legend in Table 5.

Figures 6 to [Fig pntd-0000942-g007] 8 illustrate the spatial spread of dengue fever in humans and mosquitoes through time. Each cell of the lattices in these figures corresponded to a building or a empty lot and the colors represent cell states, whose meanings are described in Table 5. The wave front is clear when human mobility is not considered (Figure 6). In the case of concentrated mobility in public locations (Figure 7), small and clear foci of disease emerge over time. As human mobility becomes more homogenous, transmission foci become less clear.

**Figure 8:**
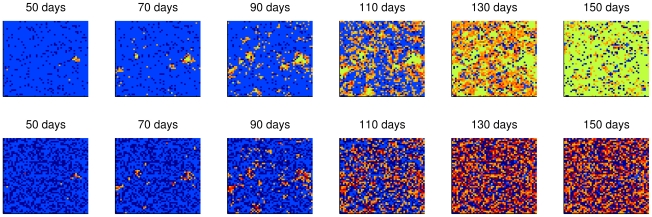
Spread pattern of dengue infection considering homogeneous human mobility. No public locations were considered: 50% of people leave home every day and visit other domiciles. Spread of infection for humans (top) and for mosquitoes (bottom). Color legend in Table 5.

**Table 5:** Color legend for the cells' states in the simulations.

	Humans	Mosquitoes
**Dark blue**	Empty lot	Without vectors
**Blue**	There is at least a susceptible human and no infected in the cell	All mosquitoes are susceptible
**Orange to red**	Increasing number of infected humans	Increasing number of infected mosquitoes
**Green**	Immunes only	-

**Figure 9:**
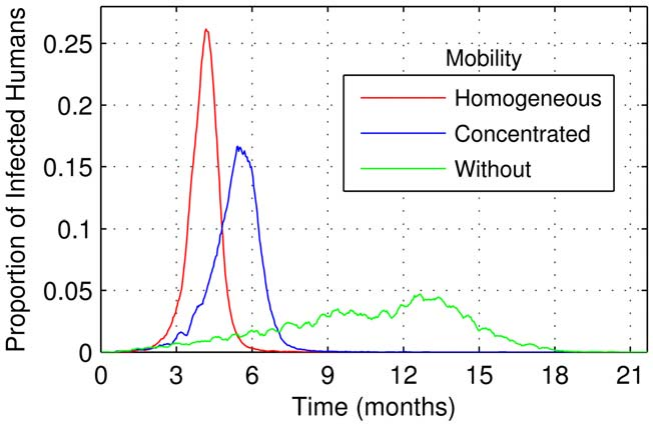
Behavior of the epidemic curves for humans, considering different configurations of human mobility.

The different propagation speeds of the disease can be observed in Figure 9. The human movement rates and patterns influence the shape of epidemic curves: the higher and more homogeneous the mobility, the higher the amplitude of the epidemic curve and more rapid its duration. Indeed, it was found that a human mobility rate *ρ_mob_* of 10% would reduce the duration of the epidemic to almost half.

### Human Renewal Rate

**Figure 10:**
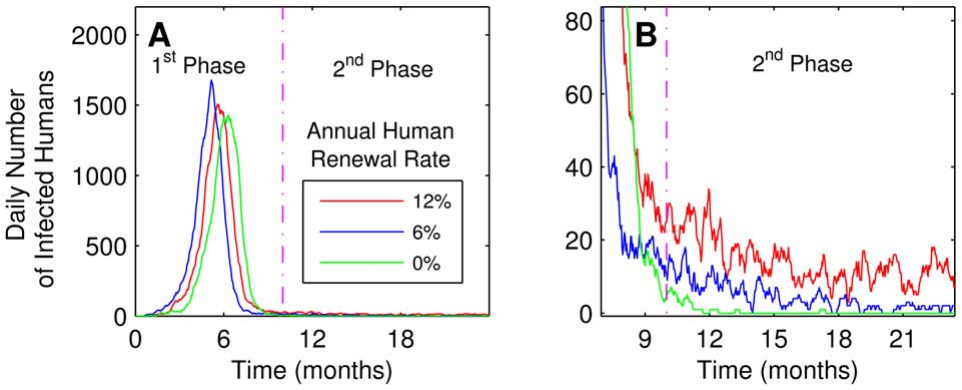
Maintenance of viral transmission in function of human renewal rate. (**A**) Two phases are clear: In the first, the epidemics with an explosion of cases and in the second, a small but persistent viral circulation. (**B**) Zoom showing that the influence of the *ρ_nh_* is clear in the second phase of the graphics.

Figure 10 shows the epidemic wave-front pattern for different annual human renewal rates *ρ_nh_* in a population with approximately 10,000 inhabitants. While for *ρ_nh_* = 0% the epidemics ended after 18 months, the viral transmission was kept active for non zero renewal rates. In fact, the amplitude of the viral transmission after the epidemic outbreak (in the second phase) was related to *ρ_nh_*. However, we found that the renewal rates had no effect in the duration and amplitude of the initial outbreak (not shown).

**Figure 11:**
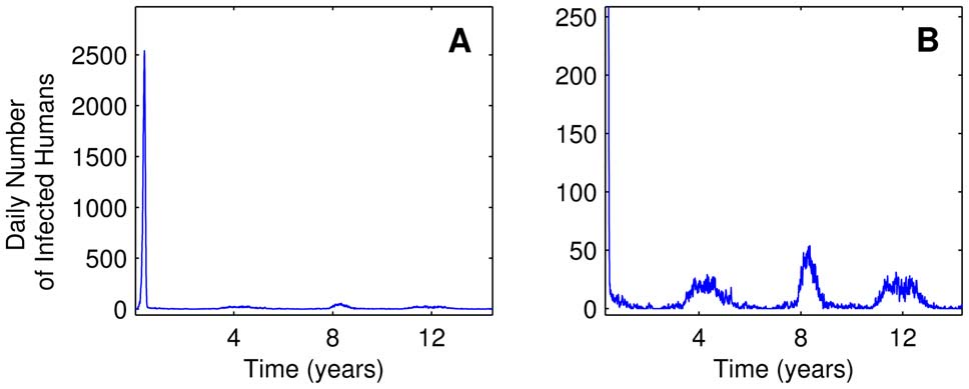
Dengue relapse for one serotype. After the epidemic (shown at the left of A), few cases sustain viral transmission (magnified in B).

The periodicity of the epidemics is shown in Figure 11. Fixing the annual human renewal rate of 3.2% in an area with 10,000 inhabitants and considering a house index of 90%, we can note the periodic behavior of the epidemics and the endemic state. After the first major epidemic, small outbreaks occur at intervals of about four years. This pattern of periodicity is consistent with patterns observed in countries of Southeast Asia and in America [1], [66], [67].

### Active Viral Transmission

**Figure 12:**
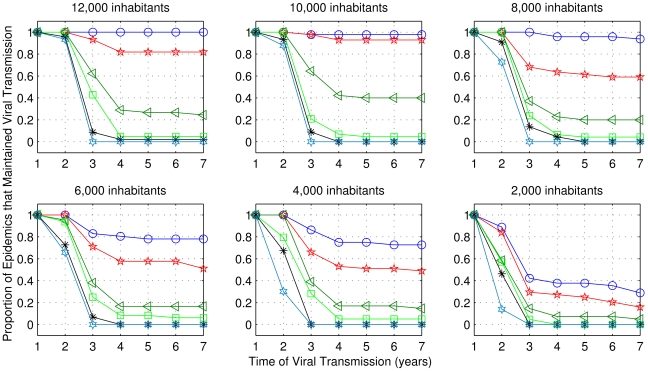
Frequency of cases with sustained viral transmission for extended periods, considering one bite per day. Simulations performed using the range of parameters described in Table 3, but for one bite per day. The values of human renewal rate are: 6%: blue; 5%: red; 4%: dark green; 3%: green; 2%: black; 1%: light blue.

**Figure 13:**
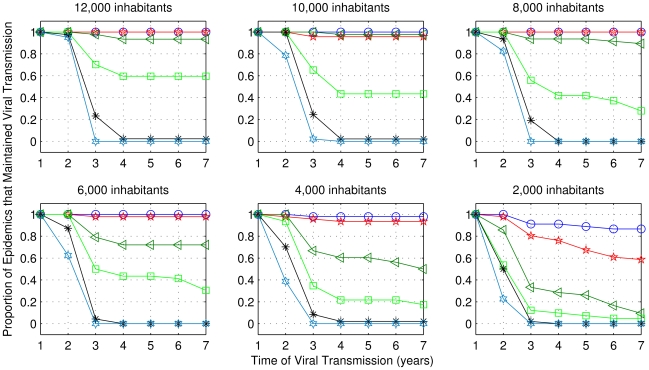
As in **Figure 12**, considering 1.5 bites per day.

For the first set of simulations, using the range of parameters described in Table 3, the percentage of replications in which an epidemic outbreak occurred and viral transmission in the first six months was over 70% in all sets of 50 replications. Figures 12 and 13 illustrate the proportion of cases, among those which the virus was transmitted in the first six months, for which transmission was maintained for a long period after the appearance of the serotype, for both biting frequencies *b_fv_* = 1 and *b_fv_* = 1.5, respectively.

The results showed that for both frequencies of bites and for all population sizes, the human renewal rate of 1% was not sufficient to maintain viral transmission for more than three years, while for 2% of human renewal, in very few cases, viral circulation was maintained for many years. The viral transmission was not sustained with the combination of small human population with low human renewal. In order to maintain viral transmission for a long period it was necessary that at least one of these parameters were not low. In the case of 8,000 inhabitants and 5% of annual human renewal rate, the chance of sustained viral circulation was higher than 50% (for both biting frequencies). Therefore, we chose these values for the second set of simulations.

**Figure 14:**
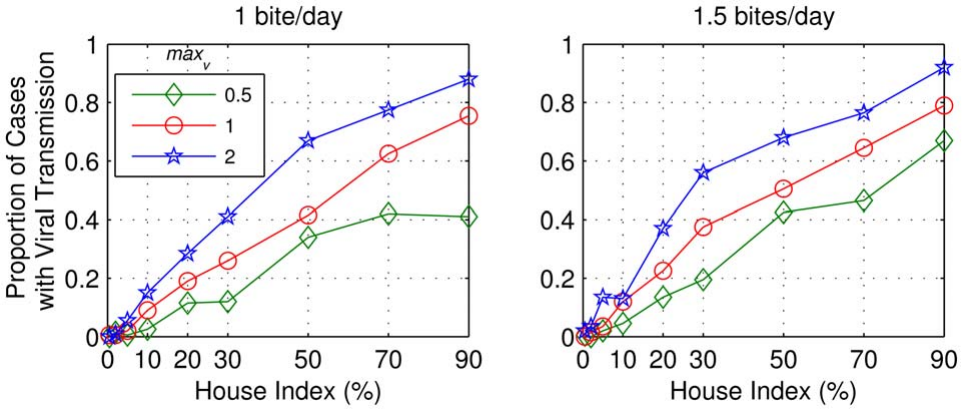
Percentage of replications which showed viral transmission in the first six months. 200 replications were performed for each combination of parameters described in Table 4, where *max_v_* means maximum ratio of vectors per human per building. Two values of mosquito daily bite rate were used: 1 and 1.5.

Figure 14 represents the percentage of cases that presented viral transmission in six months for each set of 200 replications with parameters of Table 4. The percentage of cases of viral transmission in six months decreased with decreasing house index. Nevertheless, we considered the cases with small values of house index (less than or equal to 10%) and found that viral transmission was not sustained for more than one year (not shown).

**Figure 15:**
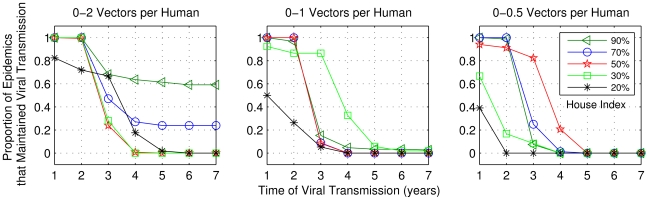
Frequency of cases among those with initial transmission, for which viral transmission was sustained. Simulations performed using the range of parameters described in Table 4, but for one bite per day. Results showed for five values of house index and three values for maximum vector/human ratio: 2, 1 and 0.5.

**Figure 16:**
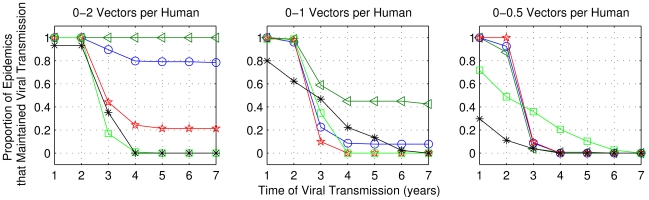
As in **Figure 15**, considering 1.5 bites per day.

Figures 15 and 16 show the percentages of cases among those with initial viral transmission, for which transmission was maintained for extended periods. The results showed that the combination of *b_fv_* = 1.5 with *max_v_* = 2 and high house index was sufficient to maintain a high probability of transmission for 7 years. However, for both frequency of bites and *max_v_*  = 1, in very few cases *it was possible* that house index between 20% and 30% maintained viral transmission active for at least five years. The results also show that the vector/human ratio influences the maintenance of viral transmission: the lower this value, the lower the viral transmission persistence.

## Discussion

Noting the limitations inherent to any mathematical modeling, we discuss the problem of viral transmission maintenance between successive epidemic periods. This question was motivated by the high incidence rates of dengue in densely populated areas of Recife [19] in 2004 and 2005. For this, we created a stochastic cellular automata model to represent the dynamics of dengue transmission in a community in which important characteristics were considered: human mobility and human renewal. Human movement transcends the spatial and temporal scales, with different influences on disease dynamics, because it influences the exposure to other individuals and thus the transmission of pathogens [44]. The simplest and traditional mathematical models for the spread of infectious diseases assume homogeneous mixing among individuals and although such models are robust, they do not reflect reality. Here we presented a non homogeneous mobility in the sense that every day most people visit public locations containing mosquitoes. Although the model considers mosquitoes in households, public locations are the main source of disease spread. We showed that human movements, concentrated or not in public locations, are responsible for the rapid development of the epidemic, reaching a very large amount of people. The other feature considered, human renewal, is responsible for the continuous increase of susceptible humans, and therefore for maintenance of viral transmission and the recurrence of outbreaks. The simulations qualitatively repeated the cyclical pattern of dengue epidemics [1], [66], [67].

With respect to the investigation of the maintenance of viral transmission for extended periods, the question to be answered was: Since the number of susceptible individuals in a naive population is virtually exhausted after an epidemic outbreak, how can the virus remain active between outbreaks? This issue was exhaustively addressed in different scenarios, where we analyzed the influence of some human and vector factors in the maintenance of viral circulation during seven years, a sufficient period for equilibrium of viral transmission [48].

The results of numerical experiments showed that with high house index values combined with high/moderate vector/human ratio, viral transmission was maintained for long periods, whereas it was not when considering the combination of small human population and low human renewal rates. The latter combination led to disease extinction in the model. Therefore, for the maintenance of viral transmission it was necessary that at least one of these parameters were not low. The extinction situation also happened when we considered house index values below 10%, for human populations with approximately 8,000 inhabitants in all cases of vector/human ratio. However, the SET model also showed that viral transmission is possible for several years (with low probability) considering low house index (between 20% and 30%), moderate ratio of vector per human (0–1 vector per person) and small human populations (approximately 4,000 people). For these cases, we believe that the random combination of factors in the initial configuration of the CA-based model allowed the virus to circulate for many years. The results of the SET model are consistent with findings from the model of Newton and Reiter [58], who concluded that viral transmission can be maintained with low house index.

**Figure 17:**
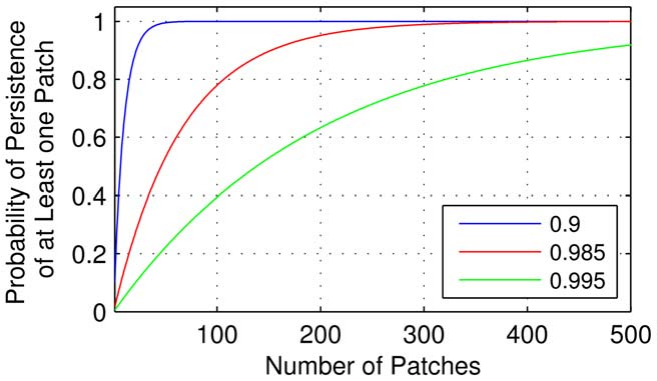
Relation between the probability of disease persistence and the number of neighborhoods *N*. Considering three values of *ρ_e_* (showed in legend), where *ρ_e_* is the probability that one of *N* independent and identical occupied patches becomes extinct in five years. The red graphic shows the case of the hypothetical example of a metropolis based on the results of SET model (Figure 15), considering neighborhoods (patches) with 8,000 inhabitants, house index of 30%, at most one vector per person, one bite per day and *ρ_e_* = 0.985.

As the neighborhoods of large cities generally have populations of at least 8,000 inhabitants, the model suggests that it is possible that in these cities a small percentage of its neighborhoods have the potential to sustain the virus for extended periods. For example, considering a hypothetical metropolis of 6 million inhabitants with house index of 30% and 750 neighborhoods of approximately 8,000 inhabitants, the SET model showed that about 1.5% of the city's neighborhoods sustain viral circulation for 5 years (or roughly 11 neighborhoods). The persistence of viral circulation is in agreement with the classic notion of extinction risk and persistence in metapopulations [68]. If *ρ_e_* is the probability that one of *N* independent and identical occupied patches becomes extinct in a certain period of time, the probability that all of them become extinct is *(ρ_e_)^N^*, thus the probability of persistence of at least one patch is *1-(ρ_e_)^N^*. For the hypothetical metropolis considered, the estimated probability for the persistence of viral transmission in 5 years was 0.015, that means *ρ_e_* = 1-0.015 = 0.985. As we have *N* = 750 neighborhoods, a number sufficiently large so that 1-(*ρ_e_*)*^N^* is nearly 1, the persistence of viral transmission in at least one neighborhood is guaranteed. To illustrate, Figure 17 shows the relation between the probability of persistence of at least one patch (disease persistence) in five years and the number of patches *N*, for three values of *ρ_e_*. In the case of *ρ_e_* = 0.985, for *N* below 15 neighborhoods (corresponding to cities with less than 120,000 inhabitants), the probability of disease extinction is high. If we had *N* = 305 neighborhoods (which corresponds to a city with 2,440,000 inhabitants), it was sufficient to guarantee 99% of chance of persistence of at least one patch. In fact, a simple analysis of the expression 1-(*ρ_e_*)*^N^* says that the greater the value of *ρ_e_*, the greater the value of *N* to ensure a high probability that at least one patch persists. This can be examined from the viewpoint of the relationship between the number of neighborhoods *N* and the combination of house index and vector/human ratio: the lower the vector infestation, the greater the value of *ρ_e_*, so the greater the value of *N* to ensure the disease persistence. The same rule applies to the converse: the greater the vector infestation, the lower the value of *N* to guarantee disease persistence. Moreover, interactions between individuals from different neighborhoods ensure a possibility of disease transmission to other districts [45]. Thus, there is a likelihood of rotation of the neighborhoods with viral circulation. This theory explains the maintenance of viral transmission in large cities.

However, in real situations, the vector population fluctuates according to a combination of meteorological factors [6]–[8], [69], [70], which modulates the number of vectors in some seasons or years, although the house index virtually does not change; Figure 1 and [19]. On the other hand, in big cities where dengue is endemic, while some districts have low infestation by vectors, others have greater abundance (thus increasing the likelihood of maintaining viral transmission for extended periods). The latter will ensure sustaining the population of mosquitoes even at low levels, despite the occurrence of seasonal variations in vector population. This occurred in some neighborhoods of the city of Recife in 2004 and 2005, where evidence showed that the vector population was not eliminated entirely by natural factors [19].

In practice, house index values should be zero or very close to zero in order to eliminate viral transmission [56], [71]. The SET model also recommends the implementation of control measures to drastically reduce the vector infestation, mainly for large cities. Moreover, the model suggests that measured house index values from field data are incorrect, since the circulation of the virus has been found even in situations with measured house index below 3% [19], [72], [73]. In a survey in a district of the city of Recife in the years 2004 and 2005 [19], a high density of *Aedes aegypti* eggs was found in the region (site 1 in part B of Figure 1), while the house index measured by health workers based on larval survey in the same neighborhood and at the same time was 0%. This apparent contradiction can be explained when considering the method of calculating the house index. The big problem with regard to the values of this index obtained from field data, is that the methodology used in most programs for controlling *Aedes aegypti*, based on larval survey, is not suitable for measuring the abundance of mosquitoes [74], disguising the true value of the house index. Thus, in agreement with Regis et al. [74], the SET model suggests that better strategies should be implemented to obtain the house index, in order to ensure better efficiency in the control programs of *Aedes aegypti*.
